# Effectiveness of ultrasound-guided fascia hydrorelease on the coracohumeral ligament in patients with global limitation of the shoulder range of motion: a pilot study

**DOI:** 10.1038/s41598-022-23362-y

**Published:** 2022-11-17

**Authors:** Hiroaki Kimura, Masei Suda, Tadashi Kobayashi, Shigeki Suzuki, Sho Fukui, Hideaki Obata

**Affiliations:** 1 Kimura Pain Clinic, Maebashi-shi, Gunma Japan; 2 Department of Rheumatology, Suwa Central Hospital, Chino-shi, Nagano Japan; 3 Immuno-Rheumatology Center, St. Luke’s International Hospital, Chuo-ku, Tokyo Japan; 4 Department of General Medicine, Hirosaki University School of Medicine & Hospital, Hirosaki-shi, Aomori Japan; 5 Center for Clinical Epidemiology, St. Luke’s International University, Chuo-ku, Tokyo Japan; 6 Department of Anesthesiology, Saitama Medical Center, Saitama Medical University, Kawagoe-shi, Saitama Japan

**Keywords:** Musculoskeletal abnormalities, Ligaments

## Abstract

We conducted a prospective single-arm interventional study of the treatment efficacy of ultrasound-guided fascia hydrorelease (US-FHR) on the coracohumeral ligament (CHL) of patients with global limitation of shoulder range of motion (ROM) without local inflammation. The primary outcome was the change in passive ROM (pROM) of external rotation (ER) after first US-FHR. Secondary outcomes included the change in pROM of other directions from baseline, the pain visual analogue scale (pVAS) at the timepoints after each procedure (first, second US-FHR and rehabilitation) as well as the change in the Shoulder Pain and Disability Index (SPADI) from the first to the second visit. Eleven patients underwent US-FHR. The pROM of ER after the 1st US-FHR changed by a median of 7.1° (*p* < 0.01). There was a statistically significant improvement in the pROM of flexion, extension, abduction, external rotation, and internal rotation from baseline to each timepoints. The pVAS at rest showed no significant improvement, although the pVAS at maximal ER showed a trend towards improvement. The SPADI score decreased by a median of 13.4 (*p* < 0.01). No adverse events were observed. US-FHR on the CHL with or without rehabilitation might be an effective, less invasive treatment for patients with global limitation of shoulder ROM.

## Introduction

Reduced shoulder range of motion (ROM) and shoulder pain of unknown cause remain substantial problem in clinical practice. Especially when the limitation of ROM is global, this condition is termed as frozen shoulder, adhesive capsulitis, or periarthritis. The boundary of this disease entity is still discussed, and several classification criteria are separately proposed^1–3^. Concerning “frozen shoulder,” the recovery from pain and restricted range of motion usually takes as much as 1–3 years, and at most 20–50% of patients suffer from persistent pain and reduced ROM^4^. The treatment for frozen shoulder includes analgesics, physical therapy, steroid injection, arthrographic distention^5^ and manipulation under anesthesia (MUA)^6^, arthroscopic capsular release^7^, and dissection of the coracohumeral ligament (CHL)/glenohumeral ligament (GHL) under arthroscopy^8^ in refractory cases. There is a strong need to develop effective and less invasive strategies.

The pathophysiology of frozen shoulder involves inflammation and the subsequent fibrotic process^9^. Microscopic observation of the affected shoulders has revealed widespread inflammation and fibrosis, not only in the capsule itself, but also in the surrounding tissue, such as the CHL, subacromial bursa, supraspinatus tendon, and subscapularis muscle^10^. In this context, frozen shoulder is more than simple “capsulitis,” and it is imperative to consider connective tissues around the joint capsule as the treatment target. Among them, the CHL plays a particularly crucial role in frozen shoulder: increased CHL elasticity^11^, thickening of the CHL as observed on ultrasound^12^, and magnetic resonance arthrography^13^ have all been reported. Furthermore, dissection of the CHL after arthroscopic capsular release leads to better recovery of ROM^8^.

Conversely, fascial dysfunction affects toward increase of stiffness in pain syndrome such as frozen shoulder^14^. Ultrasound-guided fascia hydrorelease (US-FHR) has become popular as a therapeutic options for (myo)fascial pain, especially in Japan^15^. Some reports have shown the effectiveness of US-FHR in various clinical conditions, such as myofascial pain syndrome on the shoulder^16^, acute low back pain^17^ and pain from scarring after arthroscopic knee surgery^18^. US-FHR is an injection technique for the release of a “stacking” fascia that can be visualized as hyperechoic strip-shaped lesions on ultrasound images, akin to peeling off thin stacking papers. The term “release” indicates separation in both structural or morphological views and relaxation in functional views^15^.

We hypothesized that an abnormal fascia in and around the CHL may develop as a result of the inflammation-fibrosis sequence in the patients with persistent global limitation of shoulder ROM (such as frozen shoulder). We therefore aimed to assess the treatment efficacy of US-FHR on the CHL of patients with global limitation of shoulder ROM without local inflammation.

## Outcome

The baseline characteristics of the patients are listed in Table 1. All patients were enrolled within the first year on disease onset. None of the participants had any secondary conditions such as diabetes or a history of connective tissue disease. Approximately 70% of the patients were taking analgesics (e.g., acetaminophen, Non-Steroidal Anti-Inflammatory Drugs). Notably, their pain visual analog scale (pVAS) score was relatively low at rest, while that at maximal external rotation (ER) was high.

**Table 1:** Baseline patient characteristics.

	Pt.1	Pt.2	Pt.3	Pt.4	Pt.5	Pt.6	Pt.7	Pt.8	Pt.9	Pt.10	Pt.11	Ave
Duration (mo.)	3	6	3	3	2	6	12	4	5	5	6	5.0
DM	−	−	−	−	−	−	−	−	−	−	−	
History of CTD	−	−	−	−	−	−	−	−	−	−	−	
Use of analgesics	+	+	+	+	+	−	−	−	+	+	+	
**ROM** **(degree)**												
Flex. aROM	101.8	96.8	69.2	74.2	74.8	75.2	74.6	88.5	100.0	81.6	136.6	88.5
Flex. pROM	108.0	108.1	80.0	86.1	83.6	87.0	88.1	93.0	112.6	111.6	142.5	100.0
Ext. aROM	25.1	15.0	26.1	28.3	25.9	13.5	31.3	19.1	10.4	22.4	33.4	22.8
Ext. pROM	27.1	19.1	33.0	36.8	31.7	15.5	37.8	27.5	12.5	29.7	38.0	28.0
Abd. aROM	49.0	42.9	45.1	43.5	47.5	63.3	58.9	36.3	49.5	79.9	93.3	55.4
Abd. pROM	54.5	69.7	59.2	50.5	49.6	66.4	76.8	41.6	72.3	86.4	106.6	66.7
IR. aROM	50.3	45.5	46.4	33.3	45.4	49.5	31.5	40.4	39.6	46.0	32.7	41.9
IR. pROM	50.3	49.0	53.9	35.6	52.3	53.6	35.8	49.4	45.1	48.1	44.4	47.0
ER. aROM	16.3	0.0	9.6	26.9	20.0	18.2	13.4	18.6	6.6	17.3	12.0	14.4
ER. pROM	24.5	7.8	14.1	34.3	24.6	19.9	15.6	20.6	7.3	24.7	17.4	19.1
**pVAS**												
At rest	0.0	10.0	72.0	31.0	17.0	17.0	24.0	0.0	24.0	9.0	0.0	18.5
At max. ER	78.0	46.0	88.0	96.0	90.0	99.0	52.0	60.0	83.0	61.0	77.0	75.5
SPADI	74.6	59.2	82.5	84.6	86.2	99.2	84.6	50.0	60.8	53.8	56.2	72.0

Abd, abduction; aROM, active range of motion; Ave., average; CTD, connective tissue disease; DM, diabetes mellitus; ER, external rotation; Ext, extension; Flex, flexion; IR, internal rotation; mo., month; max., maximal; pROM, passive range of motion; pVAS, pain visual analog scale; Pt, patient; SPADI, shoulder pain and disability index.

**Figure 1:**
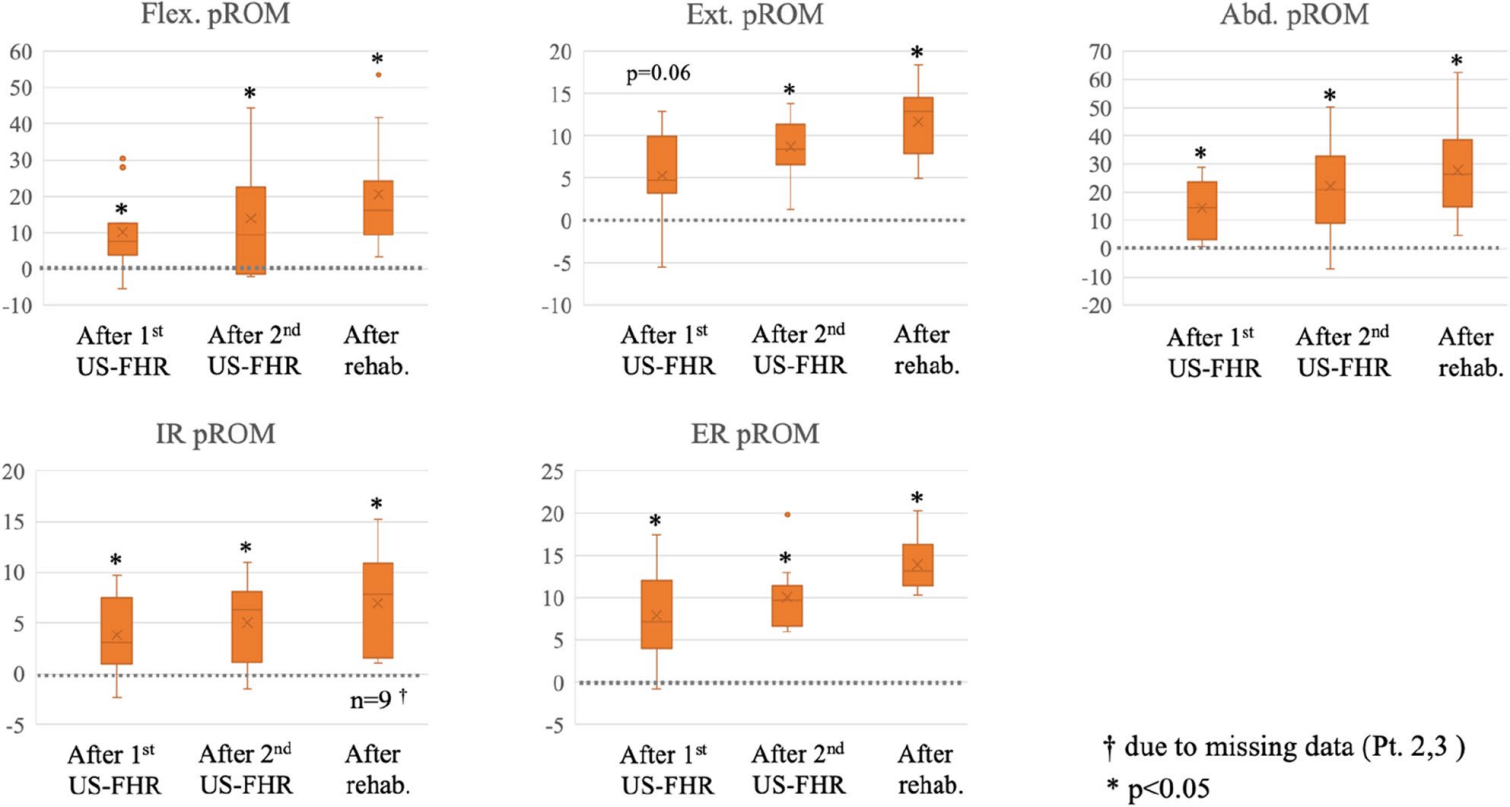
Box-and-whisker plot of ROM change over time compared to baseline. The vertical line is the measure of degree. Abbreviations: Abd, abduction; ER, external rotation; Ext, extension; Flex, flexion; IR, internal rotation; pROM, passive range of motion; rehab, rehabilitation; US-FHR, ultrasound-guided fascia hydrorelease. Note: dot lines indicate the baseline.

The median change in passive ROM (pROM) of ER from before to after the 1st US-FHR (primary outcome) was 7.1° [IQR, 4.6 to 10.6] (*p* = 0.01). The change in pROM of other directions from before to after the 1st US-FHR were as follows: flexion (median 7.7° [IQR, 5.1 to 14.4°]), extension (median 4.7° [IQR, 3.2 to 9.6°]), abduction (median 14.4° [IQR, 4.5 to 23.3°]), internal rotation (IR) (mean 3.1° [IQR, 1.8 to 6.3°]). Figure 1 shows the sequential change of pROM from before the 1st US-FHR to the end of the interventions. The change in pROM from the baseline to each timepoint (i.e.: after 1st US-FHR, after 2nd US-FHR, and after rehabilitation) was statistically significant (*p* < 0.05) in every direction except for extension (after 1st US-FHR, *p* = 0.06). The increase in pROM from baseline over time also showed statistically significant trends (*p* < 0.05) in every direction.

**Figure 2:**
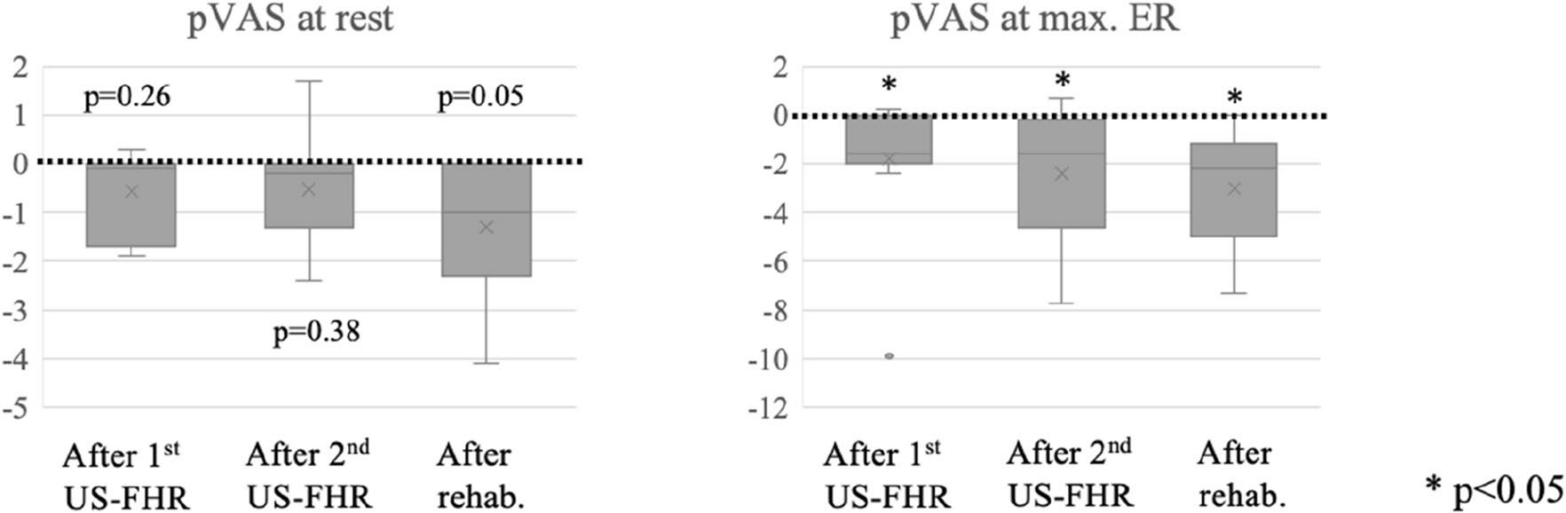
Box-and-whisker plot of pVAS change over time compared to the baseline. The pVAS at rest is scored at rest, and pVAS at max.ER is at maximal ER (on move), separately. Abbreviations: Abd, ER, external rotation; pVAS, pain visual analog scale; rehab, rehabilitation; US-FHR, ultrasound-guided fascia hydrorelease. Note: dot lines indicate the baseline.

The pVAS at rest did not show significant improvement over time, while the pVAS at maximal ER showed significant improvement at each timepoint compared to the baseline (Fig. 2, *p* < 0.05). The Shoulder Pain and Disability Index (SPADI) score changed by a median of 13.4 [IQR, 8.5 to 18.1] from baseline to the 2nd visit (*p* < 0.01).

There were no adverse events such as pain at the injection site, subcutaneous bleeding, or nerve damage related to the procedure.

## Discussion

The ROM on flexion, extension, abduction, internal rotation, external rotation remarkably improved immediately after US-FHR on the CHL. Interestingly, the ROM improvement was not limited on external rotation although we only approached the CHL. An anatomical study showed that the anterior part of the CHL envelopes the subscapularis muscle and the posterior part envelopes supraspinatus / infraspinatus muscle, anchoring the muscles to the coracoid process^19^. Therefore, we assume the change in the CHL might affect the muscles connected to CHL and improved the ROM in various aspects. The improvement after US-FHR on ROM was generally maintained throughout the consequent procedures. The total change at the end of the interventions was numerically higher than the change with the 1st US-FHR, which may support the idea that repeated US-FHR, or the combination of US-FHR and rehabilitation, may additionally improve shoulder ROM. Pain VAS at the maximal ER position showed an improving trend over time, which was not the case with VAS at rest. SPADI showed remarkable improvement between the 1st and 2nd visit. This result may indicate that US-FHR on CHL works for both pain on motion and disability of the patients with global limitation of shoulder ROM. We also observed recovery of CHL stretchability with US-FHR by dynamic ultrasound evaluation (Supplementary Video 1a and 1b), which may explain why this technique facilitates improvements of shoulder ROM. Ultrasound have been used for dynamic quantitative motion metrics^20^, and for measuring the change in elasticity before and after the treatment^21^ in various shoulder problems. Further study to measure stretchability of the CHL quantitively should be done in the future.

The mechanism of US-FHR is not fully understood yet. A previous study of US-FHR targeted the hyperechoic lesion in the multifidus muscle and assumed that the injected solution might affect pain receptors in the abnormal fascia visualized as a hyperechoic area^17^. Another possible mechanism is that releasing (separating) “stacking” fascia in the CHL increased shear strain, leading to ROM recovery. Stacking fascia (also referred as “fibrosis” of the fascia) is usually visualized as thickened or high-echoic regions on Ultrasound^22^, and a report showed these sonographic changes correlate with pain, a greater reduction in fascial shear strain, and also reduced lumber ROM in patients with low back pain^23^.

US-FHR differs from similar injection techniques in several ways: trigger point injection in myofascial pain syndrome usually does not use ultrasound and the needle tip does not target the fascia. The appropriate depth of injection for trigger point injection (TPI) is still under debate^24^. Hydrodissection separates two anatomically adjacent components with a clear border, such as a peripheral nerve and its surrounding tissue, often using a larger amount of solution^25^, whereas US-FHR injects as little as 2 mL of normal saline alone into the fascial tissue that looks thickened on ultrasound, intending to loosen and unglue it, without triggering a clear separation from the adjacent tissues. We assume that 2 mL is enough for this technique because a cadaveric study showed 1.0 mL of a pigment solution injected under ultrasound guidance separated two adjacent muscles (trapezius muscle and rhomboid muscle) and spread to the wide area within the interfascial space (24.5 cm^2^ on the deep side of the trapezius muscle and 18.8 cm^2^ on the superficial side of rhomboid muscle)^26^. Hydrodilation is sometimes used to treat frozen shoulder; this involves the injection of large amounts (often as much as 30 ml) of normal saline and steroids through the glenohumeral recess or rotator cuff interval into the shoulder capsule, leading to rupture of the capsule^27,28^. In contrast, US-FHR on the shoulder aims to release the fascial tissues outside the capsule, altering the sliding of the adjacent tissues. Prolotherapy triggers local inflammation by injecting dextrose, resulting in production of substances that promote tissue healing such as platelet-derived growth factors^29^. Contrastingly, US-FHR does not seem to affect the inflammatory process or humoral factors as ROM and pain are altered immediately after the injection.

US-FHR has several advantages over the other injection techniques. First, the procedure takes a very short time (usually only 30 s), and the results appear immediately after the procedure. Second, it uses normal saline alone, which is cost-effective and safer than other drug solutions such as corticosteroid or local anesthesia. We also favor normal saline in US-FHR, as one study showed that normal saline was more effective than mepivacaine for TPI in myofascial pain syndrome^30^. Third, ultrasound guidance enables clear visualization of the target tissue as well as the structures around, which makes the injection procedure safe. Moreover, comparing the sliding motion before and after the procedure by US informs us whether the injected target contributed to the functional impairment or not.

This study has several limitations. First, it was single-arm study and included only 11 patients due to relatively severe inclusion criteria. However, despite the small number of subjects, their ROM, pVAS at max. ER, and SPADI showed a statistically significant improving trend with US-FHR. Second, it is unclear if the effect of US-FHR lasts more than a week. Third, the response to the US-FHR varied widely among the subsects, and prediction models must be constructed to assess the efficacy.

Recently, the concept of “fascial system,” comprising the three-dimensional continuum of soft, collagen-containing, loose, and dense fibrous connective tissue that permeate the body and incorporate various type of connective tissues from adipose tissue to ligaments, has been proposed^31^. In this novel point of view, both the CHL and adjacent peribursal fat are a part of the fascial system, therefore what we have called “capsulitis” (such as frozen shoulder, adhesive capsulitis, or periarthritis) is a problem of fascial system.

In conclusion, US-FHR on the CHL with or without rehabilitation might be an effective, less invasive, and inexpensive treatment for patients with global limitation of shoulder ROM. The safety and effectiveness of the procedure should be further evaluated with controlled trials with large number of patients and longer follow-up time. We would hope that our description of this novel treatment approach targeting the fascial system surrounding the shoulder sheds new light on the pathophysiology and treatment of this condition.

## Methods

### Ethics

Ethical approval for this study was obtained from the Fukushima Medical University Ethics Committee (RK2019-002). Written informed consent was obtained from all patients. All methods were performed in accordance with the Declaration of Helsinki. This manuscript adheres to the applicable WHO recommendation.

### Patient selection

We conducted a prospective single-arm interventional study to assess the effectiveness of US-FHR in adult patients (over 20-year-old) who visited the outpatient department of the Kimura pain clinic with unilateral persistent global limitation of shoulder ROM from January 1, 2019 to June 31, 2020.

The inclusion criteria were as follows: clinical diagnosis of frozen shoulder, defined as a gradual development of global limitation of both active range of motion (aROM) and pROM of the shoulder and exclusion of differential diagnosis following the classification criteria of frozen shoulder endowed by American Academy of Orthopedic Surgeons^2^; and severe decrease of aROM and pROM (< 30% of normal ROM) on ER. Differential diagnosis were made by history of illness, physical examination and ultrasound. The sole exclusion criterion was the presence of subacromial bursitis or calcified tendonitis confirmed by ultrasound.

**Figure 3:**
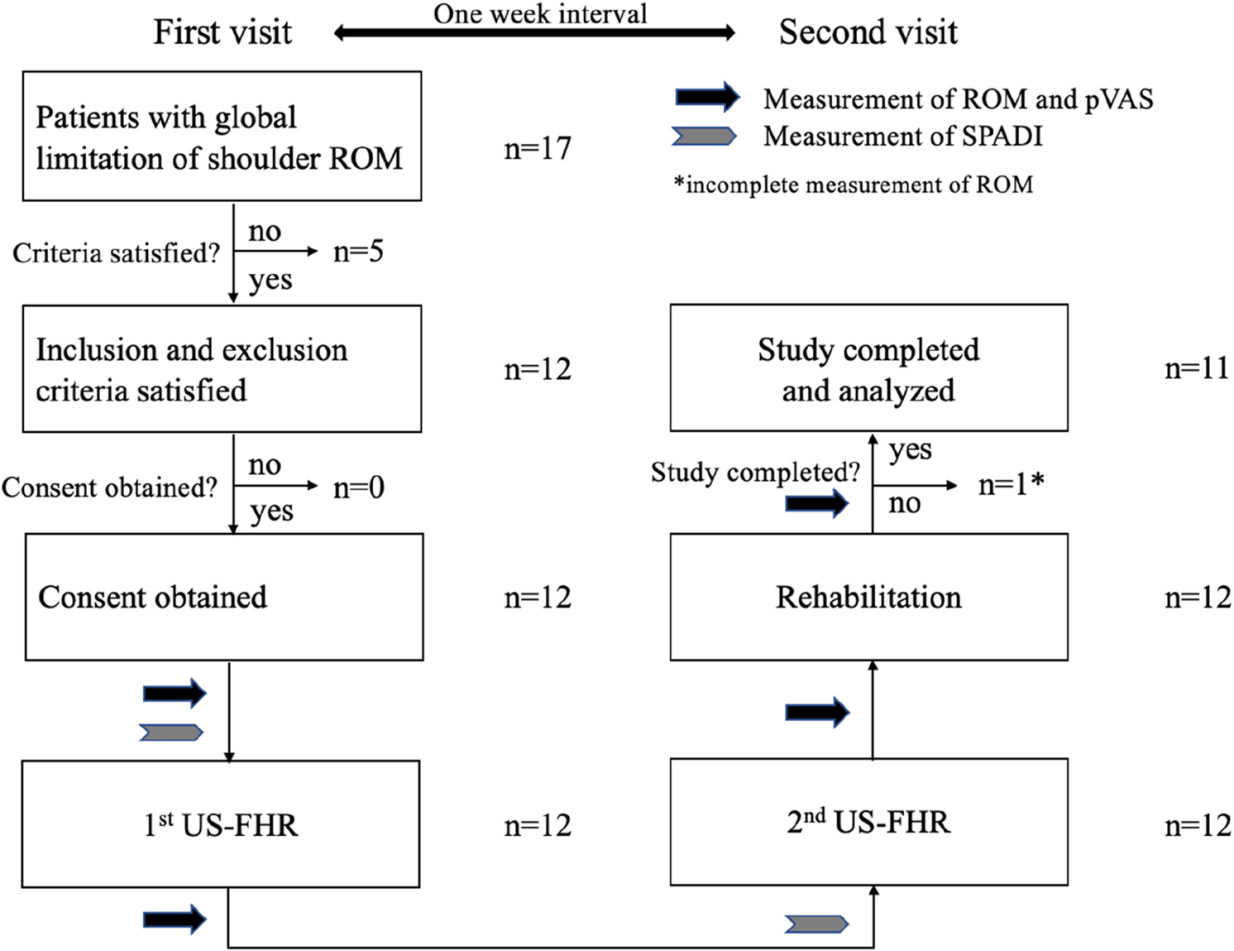
Flowchart showing patient selection and treatment. The patient’s flow was as follows: on the first visit to the clinic, the patients underwent initial assessment, informed consent process, and 1st US-FHR consecutively. On the second visit, the patients underwent 2nd US-FHR and then rehabilitation. We recruited 17 patients with global limitation of shoulder ROM. 12 of them met the inclusion and exclusion criteria. After informed consent of the study was obtained, all of the 12 underwent 1st US-FHR on the first visit, 2nd US-FHR and rehabilitation on the second visit that is scheduled approximately one week from the 1st. 11 completed the study protocol, while one did not complete the measurement and dropped out from the analysis. The black arrow indicates the time point of measuring ROM and pVAS. The gray arrowhead indicates the time point of measuring SPADI. Abbreviations: ROM, passive range of motion; SPADI, shoulder pain and disability index; US-FHR, ultrasound-guided fascia hydrorelease.

A flowchart of patient selection is shown in Fig. 3.

### Treatment

Patients underwent US-FHR on CHL on the 1st visit and 2nd visit that was scheduled around one week after the 1st. On the same day after the 2nd US-FHR, patients underwent rehabilitation that was focused mainly on the ROM training of external rotation.

**Figure 4:**
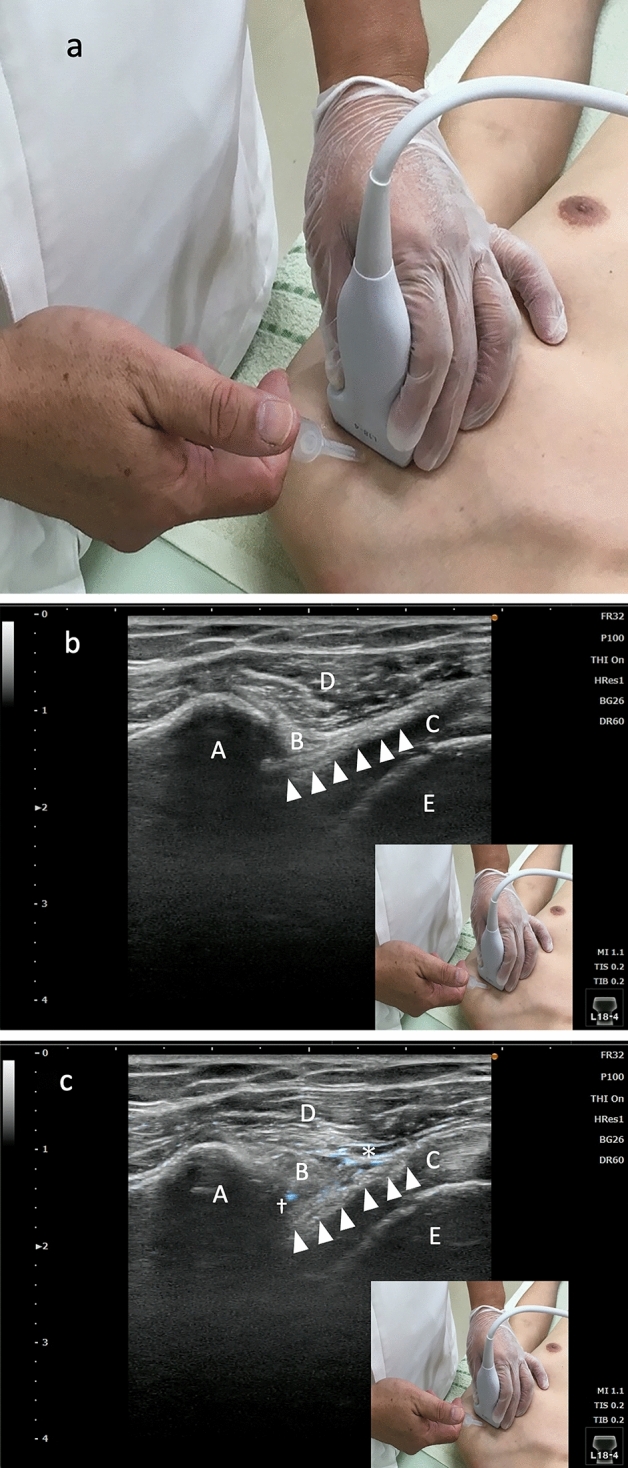
Probe position and visualization of the coracohumeral ligament (CHL). (**a**) Probe positions during ultrasound-guided fascia hydrorelease of the CHL. The patient was placed in a supine position, and the affected shoulder was maximally externally rotated within the patient’s tolerance. An ultrasound probe was placed on the upper lateral side of the shoulder. (**b**) Visualization of the coracohumeral ligament (CHL). The CHL was visualized with the probe placed at the position shown in (**a**). A, coracoid process; B, peribursal fat around subacromial bursa; C, subscapularis tendon; D, deltoid muscle; E, humeral head; arrowheads, CHL. (**c**) Ultrasound image of ultrasound-guided fascia hydrorelease (US-FHR) on the coracohumeral ligament (CHL). Two ml of normal saline was injected within the CHL. The needle tip (dagger) was always visualized and the position of the needle tip was controlled to remain on the CHL throughout the injection. The CHL becomes irregularly edematous after the US-FHR and the irregularity disappears after a certain period (depending on the subjects). A, coracoid process; B, peribursal fat around subacromial bursa; C, subscapularis tendon; D, deltoid muscle; E, humeral head; arrowhead, CHL, asterisk: fluid collection. Note: the CHL bundle was released into group of thin strip-shaped lines (the whole procedure can be viewed in Supplementary Video 2).

The procedure for USFHR is shown in Fig. 4A. Patients were placed in a neutral position. The affected shoulder was externally rotated maximally within the patient’s tolerance. We used KONICA MINOLTA HS-1 (authentication number: 226ABBZX00051000) for US-FHR. The ultrasound probe (L18-4: frequency ranges from 4 to 18 MHz [authentication number: 226ABBZX00052000]) was placed on the upper lateral side of the shoulder, and the CHL portion that bridges the coracoid process and humeral head was visualized. A 38-mm 27-gauge needle was inserted through the deltoid muscle, and the needle tip was advanced until it reached the CHL, using an out-of-plane approach (Fig. 4B). We injected 2 ml of normal saline across the bundle of the CHL (Fig. 4C), carefully monitoring the image by US. A movie of each procedure was recorded (examples are shown in Supplementary Video 2).

### Outcome measurements

The primary outcome measure was the change in the pROM of ER before and after the 1st US-FHR. The secondary outcome measure included the changes in pROM of ER from the timepoint before the 1st US-FHR to after the 1st US-FHR, after the 2nd US-FHR, and after rehabilitation, as well as the changes in pROM of other directions and the change in pVAS. The pVAS score is determined by measuring the distance (mm) on the 100 mm line between the “no pain” anchor and the patient’s mark with a ruler, providing a range of scores from 0 to 100. pVAS at rest and at maximal ER (on move) were scored separately. SPADI from the 1st visit to 2nd visit (surveyed before US-FHR on each visit) was also included in the secondary outcome measure. The ROMs were measured with digital goniometer (produced by Almencla US) twice by a well-experienced physical therapist, and the average value was calculated. Before clinical use, the accuracy of the device was checked by comparing the measured value with that of the analogue goniometer at 45° and 90°.

Use of analgesics was allowed during the study period; however, nerve root block, steroid injection on the shoulder joint, acupuncture, and rehabilitation were not allowed during the time between the 1st and the 2nd visit.

### Statistical analysis

Categorical and quantitative variables were described as numbers (percentages), means (standard deviation) or medians (interquartile range [IQR]), as appropriate. For each ROM, the measured value at each assessment point after the procedure (after the 1st US-FHR, before and after the 2nd US-FHR, and after the rehabilitation) were compared with those at baseline (before the 1st US-FHR) using Wilcoxon-signed rank test. *p* values were corrected by the Bonferroni method based on the number of multiple testings. In addition, trends for the improvement in each ROMs were evaluated using the Jonckheere-Terpstra trend test. Differences in SPADI between baseline and on the 2nd visit were assessed by Wilcoxon-signed rank test. For all analyses, a *p* value < 0.05 was considered significant. All analyses were performed using STATA software (version 17.0, StataCorp, College Station, TX, USA).

## Supplementary Information

Supplementary Video legends.

Supplementary Video 1a.

Supplementary Video 1b.

Supplementary Video 2.

## Data Availability

The authors confirm that the data supporting the findings of this study are available within the article and its supplementary materials.
