# Supplementary material for: Effectiveness of ultrasound-guided fascia hydrorelease on the coracohumeral ligament in patients with global limitation of the shoulder range of motion: a pilot study
Source: Sci Rep. 2022 Nov 17;12:19782. doi: 10.1038/s41598-022-23362-y (PMC9671893; doi:10.1038/s41598-022-23362-y)
Supplement: Supplementary file 1 — Supplementary Video legends. [file 41598_2022_23362_MOESM1_ESM.docx]

**Supplementary Digital Content**

Supplementary video 1. Sonographic changes in the coracohumeral ligament (CHL) sliding movement before and after ultrasound-guided fascia hydrorelease (US-FHR)

(a) Before US-FHR

(b) After US-FHR

Note: the CHL is remarkably more stretched due to increased extensibility.

Supplementary video 2. Ultrasound movie of ultrasound-guided fascia hydrorelease on the coracohumeral ligament

Note: The blue light indicates movement (that is, fluid that is being injected, or moving tissue and needle tip)
